# Stabilization of Fully Deprotonated Melaminate Anions (C_3_N_6_)^6–^ in M_3_(C_3_N_6_) (M = Cd, Ca)

**DOI:** 10.1021/jacs.5c16752

**Published:** 2026-01-15

**Authors:** Pascal L. Jurzick, Lukas Brüning, Björn Winkler, YiXu Wang, Richard Dronskowski, Elena Bykova, Dominik Spahr, Michael Hanfland, Björn Wehinger, Nico Giordano, Maxim Bykov

**Affiliations:** a Institute of Inorganic and Analytical Chemistry, 9173 Goethe University Frankfurt, Max-von-Laue-Straße 7, 60438 Frankfurt am Main, Germany; b Institute of Geosciences, 9173 Goethe University Frankfurt, Altenhoeferallee 1, 60438 Frankfurt am Main, Germany; c Chair of Solid-State and Quantum Chemistry, Institute of Inorganic Chemistry, RWTH Aachen University, 52056 Aachen, Germany; d 55553 European Synchrotron Radiation Facility, 38043 Grenoble, France; e 28332 Deutsches Elektronen-Synchrotron DESY, Notkestraße 85, 22607 Hamburg, Germany

## Abstract

Hydrogen-free melaminate salts M_3_(C_3_N_6_) (M = Cd, Ca) were synthesized in laser-heated diamond anvil cells at 34–48 GPa and 2000–2500 K. Cd_3_(C_3_N_6_) was synthesized via a direct reaction between the elements, while Ca_3_(C_3_N_6_) was obtained following a rational chemical design approach from calcium carbodiimide, Ca­(NCN), which served as a single-source precursor. Both compounds contain the fully deprotonated melaminate anion (C_3_N_6_)^6–^, representing a fundamental milestone in nitridocarbonate chemistry. The crystal structures of M_3_(C_3_N_6_) were solved and refined using synchrotron single-crystal X-ray diffraction data and were fully corroborated by density functional theory calculations. Cd_3_(C_3_N_6_) crystallizes in the acentric *R*3*c* and Ca_3_(C_3_N_6_) in the centrosymmetric *R*3̅*c* space groups, and both compounds are recoverable to ambient conditions. Extending this design principle, our calculations indicate that Zn and Pb melaminates are thermodynamically accessible under similar conditions, highlighting the general stability of hydrogen-free nitridocarbonates of selected divalent metals.

## Graphical abstract

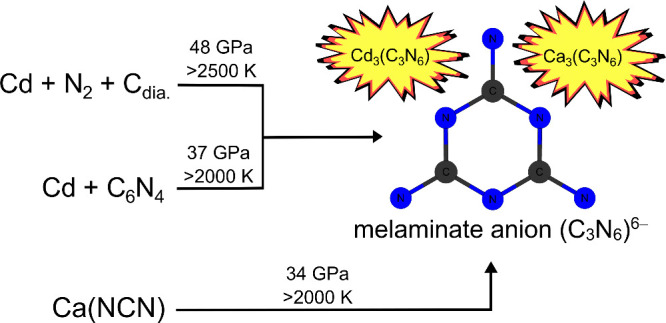

## Introduction

Molecular melamine (C_3_H_6_N_6_) and its derivative compounds serve as fundamental building blocks in the synthesis of melamine-formaldehyde resins or flame-retardant materials.^1,2^ Moreover, it is one of the main precursors for producing graphitic carbon nitride (*g*-C_3_N_4_), which is studied for its high-performance photocatalysis and optoelectronics applications.^3^ The physicochemical properties of *g*-C_3_N_4_ can be tuned by incorporating different metals.^4–7^ For instance, lithium or potassium ions can act as structure-directing agents during the formation of the porous frameworks, influencing photocatalytic activity.^6,7^ Despite a variety of synthetic routes to *g*-C_3_N_4_, the ideal composition C_3_N_4_ is almost never reached, but instead, one finds partially amorphous phases, often contaminated with hydrogen and oxygen; likewise, there is no convincing single-crystal structure model yet. A rational design route toward pure *g*-C_3_N_4_ is to start by synthesizing its hydrogen-free building blocks. The most straightforward metathetic approach to substituted hydrogen-free nitridocarbonates is well established for acidic precursors (e.g., HCN) but becomes increasingly more difficult for basic precursors containing larger numbers of hydrogen atoms.

For example, in the case of guanidine CNH­(NH_2_)_2_, usually only single or double deprotonation can be achieved, yielding compounds M­(CN_3_H_4_)_2_ (M = Eu, Ba)^8,9^ and MC­(NH)_3_ (M = Ca, Sr, Eu, Yb).^9–12^ The first examples of stabilizing the fully deprotonated guanidinate anion (CN_3_)^5–^ were reported in compounds SbCN_3_ ^13^ and Ln_3_O_2_(CN_3_) (Ln = La, Eu, Gd, Tb, Ho, Yb).^14^ In both cases, laser-heated diamond anvil cells (LHDACs) were used to synthesize and study the reaction products *in situ*, revealing the importance of hydrogen-free synthesis conditions. Stelzer et al. have produced deprotonated (CN_3_)^5–^ stabilized in (Sr_9_N_1.33(8)_)­(SrIn_3_)­[CN_3_] and Sr_4_(Sr_6_N)_2_­[In_4_]­[CN_3_]_4_ compounds without application of nitrogen pressure from a sodium flux.^15^

Complete deprotonation of the next members of the nitridocarbonate series becomes increasingly challenging. In 1922, Franklin had reported the synthesis of melaminate salts KC_3_N_6_H_5_·NH_3_ and K_3_C_3_N_6_H_3_ in liquid ammonia from melamine and potassium amide, based on elemental analysis.^16^ The confirmation of single-deprotonated melaminates via single-crystal X-ray diffraction (scXRD) was reported by Görne et al. in 2021 with the potassium and rubidium compounds KC_3_N_6_H_5_·NH_3_ and RbC_3_N_6_H_5_·^1^/_2_NH_3_.^17^ Based on IR spectroscopy, they also obtained NaC_3_N_6_H_5_·*n*NH_3_ and K_3_C_3_N_6_H_3_,^17^ which was the proposed reaction product by Schnick et al. in 1995 as well.^16,18^ The melaminate anion (C_3_H_3_N_6_)^3–^ was reported in 2021 by Kallenbach et al. with the synthesis of a Metal–Organic Framework containing dehydrogenated melamine.^19^ The synthesis of tricopper­(I) melaminate Cu_3_(C_3_H_3_N_6_) was achieved by a solid-state reaction of CuCl with melamine under flowing argon at 275 °C. Recently, Bayat et al. observed the same reaction product by a reaction of CuCl and sodium hydrogen cyanamide Na­(HCN_2_), forming Cu_3_(C_3_N_6_H_3_) and NaCl.^20^ Double-deprotonated melaminate was synthesized in 2023, also by Bayat et al., in a solid-state reaction of antimony­(III) chloride SbCl_3_ and melamine, which led to the formation of SbCl­(C_3_N_6_H_4_) and (C_3_N_6_H_7_)­Cl.^21^ The summary of synthetic routes to substituted melaminate salts is given in Table S1.

Recently, Chen et al. proposed a synthetic route to the completely deprotonated melaminate salt of the composition WC_3_N_6_.^22^ The metathetic pathway involves a reaction between tungsten oxide (WO_3_) and melamine (C_3_H_6_N_6_), forming hydrogen-free tungsten melaminate (WC_3_N_6_) and gaseous water (H_2_O).^22^ Calculations show that both predicted polymorphs of WC_3_N_6_ are indirect semiconductors, with electrical and optical properties, suitable for photocatalysis and optoelectronic devices.^22^ During the preparation of this manuscript, the high-pressure synthesis of hydrogen-free lead melaminates *hP*72-Pb_3_(C_3_N_6_) and *tP*48-Pb_3_(C_3_N_6_) has been reported.^23^

## Results and Discussion

Here, we present the stabilization of a fully deprotonated melaminate (C_3_N_6_)^6–^ anion in a series of salts of divalent metals M_3_(C_3_N_6_) (M = Ca, Cd) in several independent experiments using various precursors (Table 1). Cadmium melaminate Cd_3_(C_3_N_6_) was synthesized in a LHDAC in two independent experiments in a pressure range 44–48 GPa (Table 1). In experiment #1 the synthesis was performed directly from the elements. A piece of Cd was placed on a diamond culet, and the sample chamber was filled with nitrogen, which served both as a pressure-transmitting medium and as a reactant. The DAC was compressed to the target pressure, and then a focused Nd:YAG laser (λ = 1064 nm) was used to heat the Cd piece. The products were then studied by means of synchrotron X-ray diffraction at the ESRF (ID15b and ID27 beamlines). The Supporting Information Section B provides complete experimental details.

**Table 1:** Summary of Experimental Conditions for the Synthesis of M_3_(C_3_N_6_) (M = Cd, Ca)

experiment	reagents	pressure, GPa	beamline
#1	Cd + C_dia_ + N_2_	48	ESRF ID15b
#2	Cd + C_6_N_4_	44	ESRF ID27
#3	Ca(NCN)	34	DESY P02.2, ESRF ID27

The diffraction patterns of the heated samples revealed the presence of numerous single-crystalline grains in the sample chamber, and the data sets were analyzed using the well-established procedures for handling multigrain samples using Domain Auto Finder (DAFi) program.^24^ Based on this analysis, several most prominent grains could be indexed with the *R*-centered hexagonal unit cell with *a*, *b* = 11.535(1) Å and *c* = 5.189(2) Å for one of such grains at 48(1) GPa (see Figure S1 and Tables S2 and S4–S8 for full details). The structure solution and refinement revealed the chemical formula of the new compound as Cd_3_(C_3_N_6_) crystallizing in space group *R*3*c* (Figure 1). In addition to this compound, we also found cadmium diazenide CdN_2_, which will be reported elsewhere. To enhance the synthetic strategy and avoid the formation of CdN_2_, we used tetracyanoethylene (C_6_N_4_) in experiment #2 as a precursor of carbon and nitrogen for the synthesis of Cd_3_(C_3_N_6_). In this experiment, C_6_N_4_ also served as a pressure-transmitting medium (Figure S2). Laser-heating at 44(1) GPa once again led to the formation of Cd_3_(C_3_N_6_), as confirmed by scXRD as well as Raman spectroscopy (Figure 1). The sample was decompressed stepwise, allowing the collection of scXRD data at each pressure point down to 30(1) GPa (Figure 2). Powder XRD from Cd_3_(C_3_N_6_) crystallites could be traced down to atmospheric pressures (Figure S3), indicating recoverability to ambient conditions.

**Figure 1:**
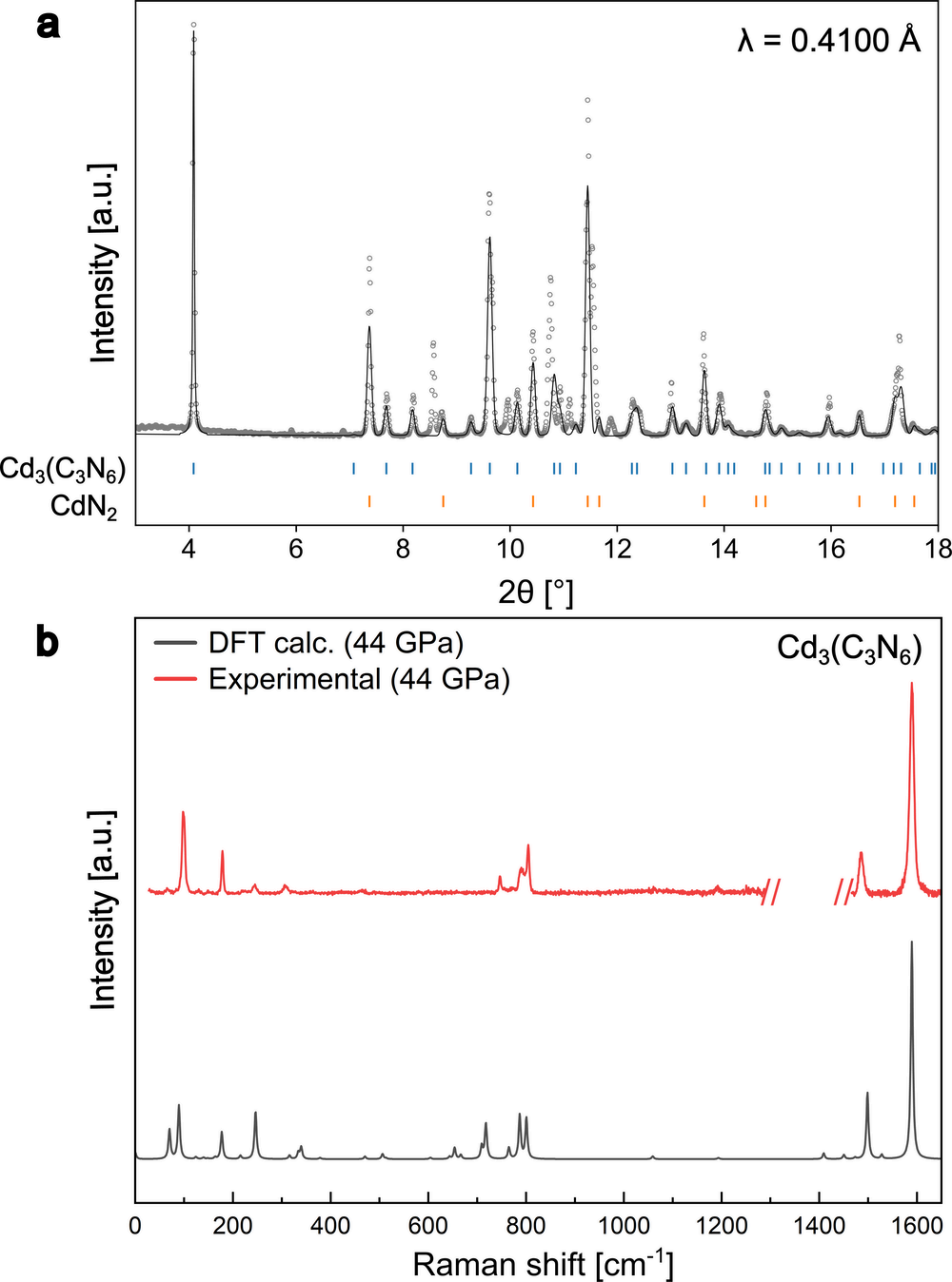
Powder X-ray diffraction and Raman spectroscopy data for Cd_3_(C_3_N_6_). (a) Le Bail fit from multigrain Cd_3_(C_3_N_6_) at 38(2) GPa compared with calculated peak positions for Cd_3_(C_3_N_6_) and CdN_2_. Structure refinements and phase identification were based on single-crystal data sets. (b) Raman spectrum of Cd_3_(C_3_N_6_) at 44(1) GPa compared with calculated Raman spectrum of Cd_3_(C_3_N_6_). Calculated Raman spectrum frequencies were scaled by a factor of 1.007, and intensities were normalized using the most intense peak as a reference.

**Figure 2:**
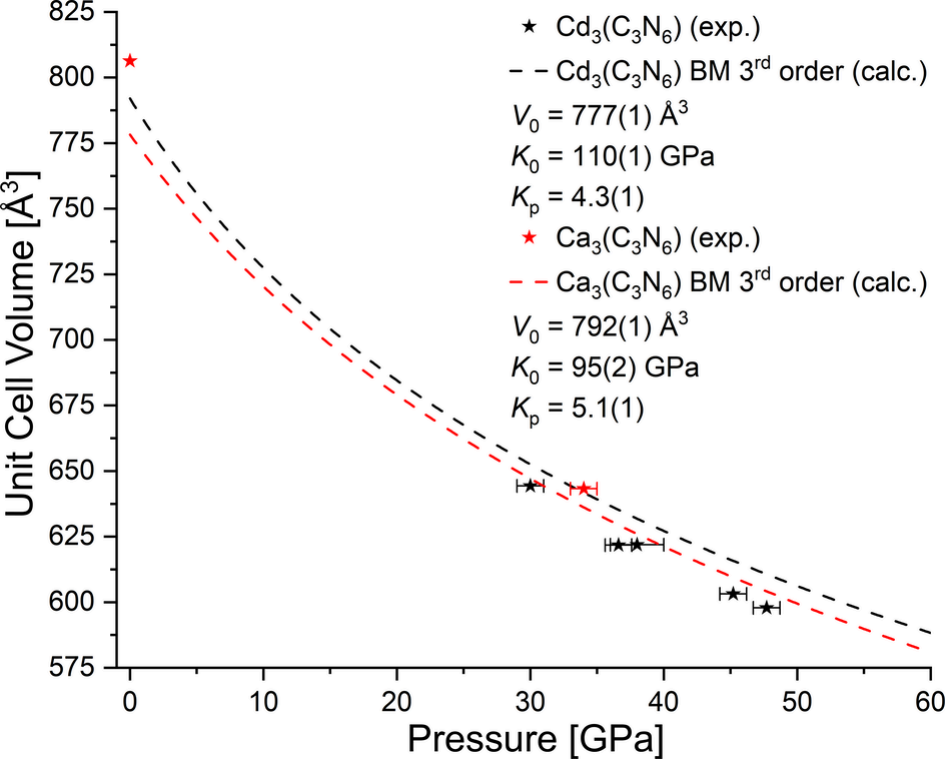
Compressional behavior of M_3_(C_3_N_6_) (M = Cd, Ca). Shown are the experimental (black and red stars) and calculated (black and red dashed line) unit cell volumes of M_3_(C_3_N_6_). The 3rd order Birch–Murnaghan equation of state was used to determine the bulk moduli of M_3_(C_3_N_6_) based on the calculated data.

The crystal structure of Cd_3_(C_3_N_6_) contains one crystallographically independent Cd, one C, and two N atoms all occupying Wyckoff sites 18*e* (see Tables S2 and S4–S8 for complete refinement details). The main structural feature of the compound is a melaminate anion (C_3_N_6_)^6–^ as shown in Figure 3. The anion is slightly distorted out-of-plane and there are three inequivalent, but similar within the standard uncertainties, C–N distances in the range 1.32–1.35 Å, demonstrating a significant degree of π-electron delocalization, which is consistent with protonated melamine itself.^25^ Melaminate anions have two resonance form types: one where the aromatic system in the ring persists and the nitrogen atoms of the amine groups are sp^3^ hybridized, and another where one or more of the amine nitrogen atoms donate a lone pair and become sp^2^ hybridized to create a double bond and thus become part of the conjugated system. Note that the sp^3^ and sp^2^ designators refer to the simplistic valence-bond model.

**Table 2:** Experimental and Calculated C–N Bond Distances within the Melaminate Anions^a^

compound	C–N bond length (*x*), Å	C–N bond length (*y*), Å	C–N bond length (*z*), Å	pressure (GPa)
Cd_3_(C_3_N_6_)	1.388^[a]^	1.338^[a]^	1.380^[a]^	0.0001
	1.347^[a]^	1.318^[a]^	1.339^[a]^	38
	1.344(33)	1.322(21)	1.347(31)	38(2)
Ca_3_(C_3_N_6_)	1.396(6)	1.329(10)	1.397(8)	0.0001
	1.353^[a]^	1.302^[a]^	1.353^[a]^	35
	1.353(5)	1.303(3)	1.352(5)	34.4(10)

a Notes: ^[a]^ marks calculated bond lengths. The geometry of the anion and labeling of the C–N bonds (*x*, *y*, *z*) are shown in Figure 3e.

**Figure 3:**
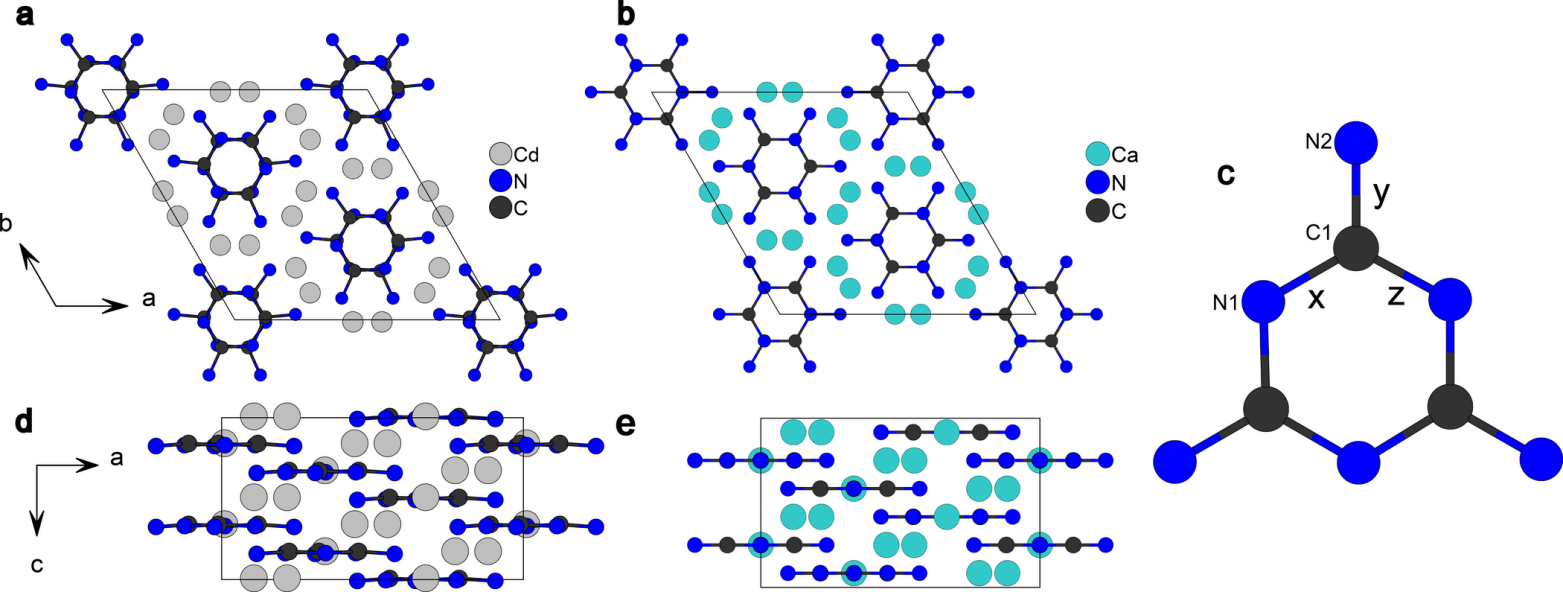
Crystal structures of Cd_3_(C_3_N_6_) at 38(2) GPa and Ca_3_(C_3_N_6_) at 34.4(10) GPa. (a) A view along the crystallographic *c*-axis of Cd_3_(C_3_N_6_). (b) A view along the crystallographic *c*-axis of Ca_3_(C_3_N_6_). (c) Melaminate anion (C_3_N_6_)^6–^. *x*, *y*, and *z* denote distinct bond distances presented in Table 2. (d) A view along the crystallographic *b*-axis of Cd_3_(C_3_N_6_). (e) A view along the crystallographic *b*-axis of Ca_3_(C_3_N_6_).

The melaminate anion (C_3_N_6_)^6–^ perfectly satisfies the charge balance in Cd_3_(C_3_N_6_), where Cd exhibits an oxidation state of +II. The empirical formula of cadmium melaminate, CdCN_2_, corresponds to the empirical formula of cadmium carbodiimide. This stoichiometric match suggests that single-source carbodiimide precursors could be used for the production of melaminate salts. To prove this hypothesis, we used Ca­(NCN) as a single-source precursor for the synthesis of Ca_3_(C_3_N_6_). In this experiment, phase-pure Ca­(NCN) was loaded in a DAC without any pressure-transmitting medium and laser-heated at 34.4(10) GPa using a CO_2_ laser (λ = 10 600 nm) (Table 1, Figure S4). The diffraction patterns and additional Raman spectra were collected and analyzed in a manner similar to that for Cd_3_(C_3_N_6_) (Figures 4 and S5). Structure solution and refinement revealed the formation of calcium melaminate Ca_3_(C_3_N_6_) crystallizing in the centrosymmetric space group *R*3̅*c* (see Tables S3 and S9, S10 for full details). The difference between the crystal structures of Ca_3_(C_3_N_6_) and Cd_3_(C_3_N_6_) is the rotation of melaminate groups with respect to each other in the neighboring layers (Figure 3), as well as the significant degree of out-of-plane distortion of melaminate anions in the latter compound (Figure 5). According to our density functional theory (DFT) calculations and experimental results, the relative rotation of melaminate anions in Cd_3_(C_3_N_6_) correlates with both the out-of-plane distortion of the melaminate units and the applied pressure (Figure S6). It should be noted that we have performed structure solution and refinement in both space groups (*R*3̅*c* and *R*3*c*) for both Ca_3_(C_3_N_6_) and Cd_3_(C_3_N_6_) compounds. In the case of Cd_3_(C_3_N_6_), it was not possible to obtain a reasonable structure refinement in a centrosymmetric *R*3̅*c* space group. In the case of Ca_3_(C_3_N_6_), the refinements in *R*3*c* and *R*3̅*c* resulted in nearly identical agreement factors, which allows us to prefer the *R*3̅*c* model. This assignment is also supported by our DFT calculations and the crystallographic structure validation algorithms.

**Figure 4:**
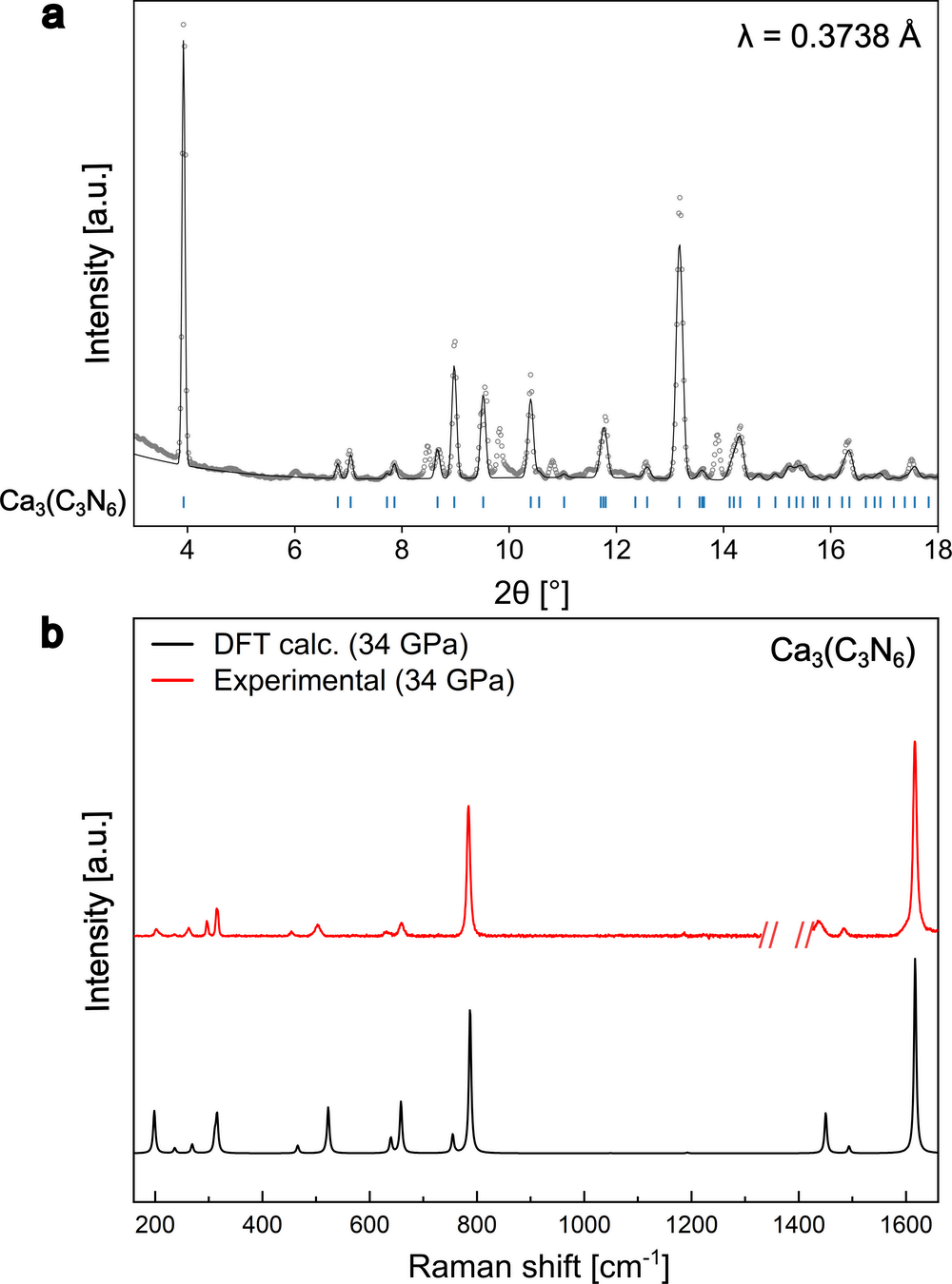
Powder X-ray diffraction and Raman spectroscopy data. (a) Le Bail fit from multigrain Ca_3_(C_3_N_6_) at 34.4(10) GPa compared with calculated peak positions for Ca_3_(C_3_N_6_). Structure refinements and phase identification were based on single-crystal data sets. (b) Raman spectrum of Ca_3_(C_3_N_6_) at 34.4(10) GPa compared with calculated Raman spectrum of Ca_3_(C_3_N_6_). Calculated Raman spectrum frequencies were scaled by a factor of 1.008, and intensities were normalized by using the most intense peak as a reference.

**Figure 5:**
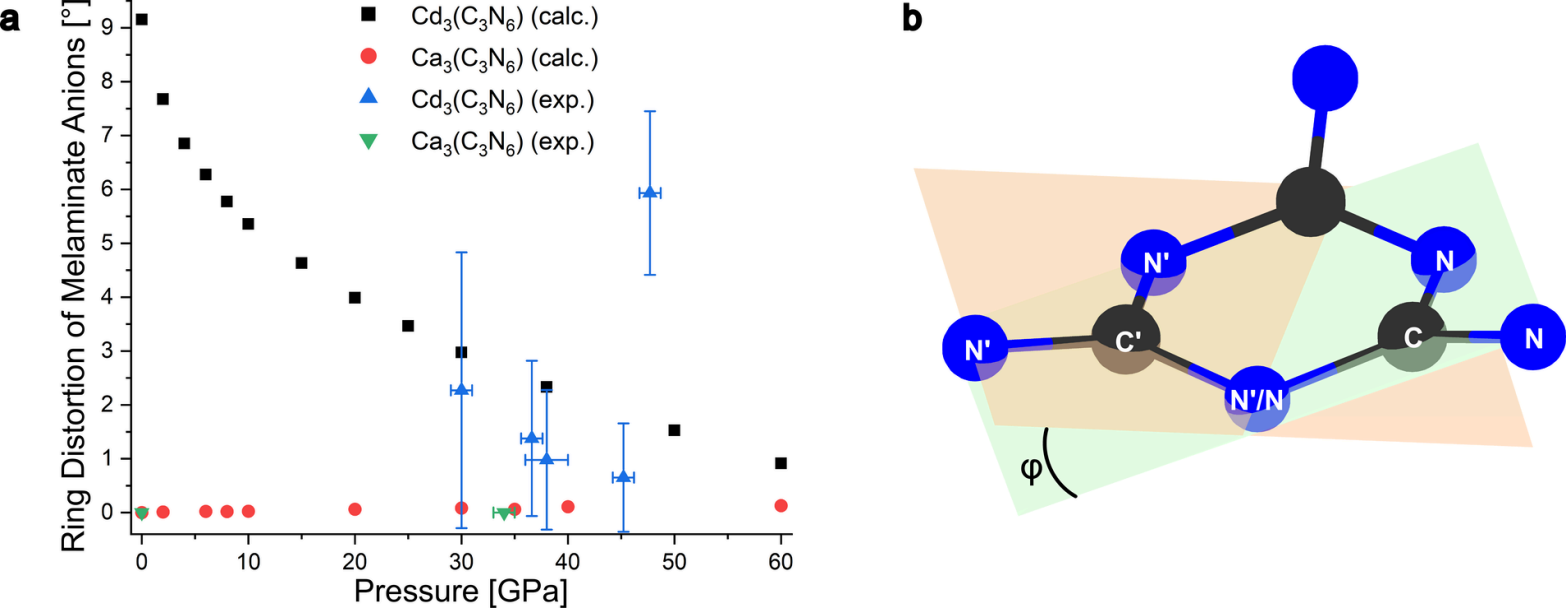
(a) Ring distortion of melaminate anions (C_3_N_6_)^6–^ in M_3_(C_3_N_6_) (M = Cd, Ca). The distortion is indicated by the angle φ between the CNNN and C′N′N′N′ planes as shown in (b). At the highest-pressure point, the atom quartets defining the planes (CNNN and C′N′N′N′) become nonplanar themselves, which might indicate further structural distortion.

To get a deeper insight into the electronic structures of the synthesized compounds, we performed theoretical calculations within the framework of the plane-wave DFT which was then unitarily transformed to local orbitals (LOBSTER) to allow for chemical-bonding analysis.^26–30^ Full details are provided in the Supporting Information. First, geometry-optimized crystal structures of the synthesized compounds are in good agreement with the experimental data, and our calculations properly reproduce the distortion of the melaminate groups in Cd_3_(C_3_N_6_). The reason for that distortion is the covalency of Cd–N bonds, which imposes geometric constraints on the rings. Wiberg–Mayer bond orders for the Cd–N bonds range between 0.30 and 0.35, while for the far less covalent, essentially ionic Ca–N bonds, the numbers are smaller, 0.06–0.18. One can draw an analogy with azides: Cd­(N_3_)_2_ has a distorted N_3_^–^ anion while it is less distorted in Ca­(N_3_)_2_.^31,32^ Similar anionic distortions have also been observed in covalent carbodiimides and cyanamides, such as Pb­(NCN).^33^ There are significantly different anionic C–N distances (1.16 Å and 1.30 Å) in Pb­(NCN), mirroring the greater covalency of Pb–N bonds, while C–N distances are of equal length (1.22 Å) in ionic Ca­(NCN).^33^

For detailed chemical-bonding analysis of Ca­(NCN) and Ca_3_(C_3_N_6_) we recalculated the crystal orbital bonding index (COBI) of all C–N bonds. The energy-resolved COBI results are presented in Figure 6a,b. The Fermi level (ε_F_) nicely separates bonding (below ε_F_) and antibonding (above ε_F_) levels for Ca­(NCN), but one recognizes a few tiny populated antibonding levels below ε_F_ in Ca_3_(C_3_N_6_), which explains the necessity of applied pressure to stabilize this compound similar to a variety of high-pressure dinitrides.^34,35^ The bond order can be quantified by the energy integral of COBI (ICOBI), which is also presented in Figure 6. In Ca­(NCN), the ICOBI sum reached 11.286 going back to six C–N bonds, so the bond order is 11.286 ÷ 6 = 1.88 ≈ 2, very close to a C=N double bond. For Ca_3_(C_3_N_6_) the C–N ICOBI values arrive at 4.236 (for three terminal C–N bonds) and 6.786 (for six in-ring C–N bonds), so the terminal C–N bonds come out stronger (bond order 1.412), and the in-ring C–N bonds are weaker (bond order 1.131), in approximate agreement with the idealized bond orders derived from valence-bond theory (1.44 and 1.28, respectively). The sum over all C–N bonds is 11.022, so covalency has decreased by a small (2%) amount compared to the prior 11.286 for Ca­(NCN). To compensate for that, the Löwdin charges (see Table S21) show that the cationic Ca charge also decreases upon pressure increase, while the cationic C/anionic N charges increase, so opposite effects are at play. Given the smaller volume, however, the calculated Madelung energies based on Löwdin charges and interatomic distances increase from Ca­(NCN) (−6.36 MJ mol^–1^) to Ca_3_(C_3_N_6_) (−6.73 MJ mol^–1^), so the overall ionicity is significantly enhanced upon forming Ca melaminate (by ca. 6%), making Ca_3_(C_3_N_6_) more salt-like compared to Ca­(NCN).

**Figure 6:**
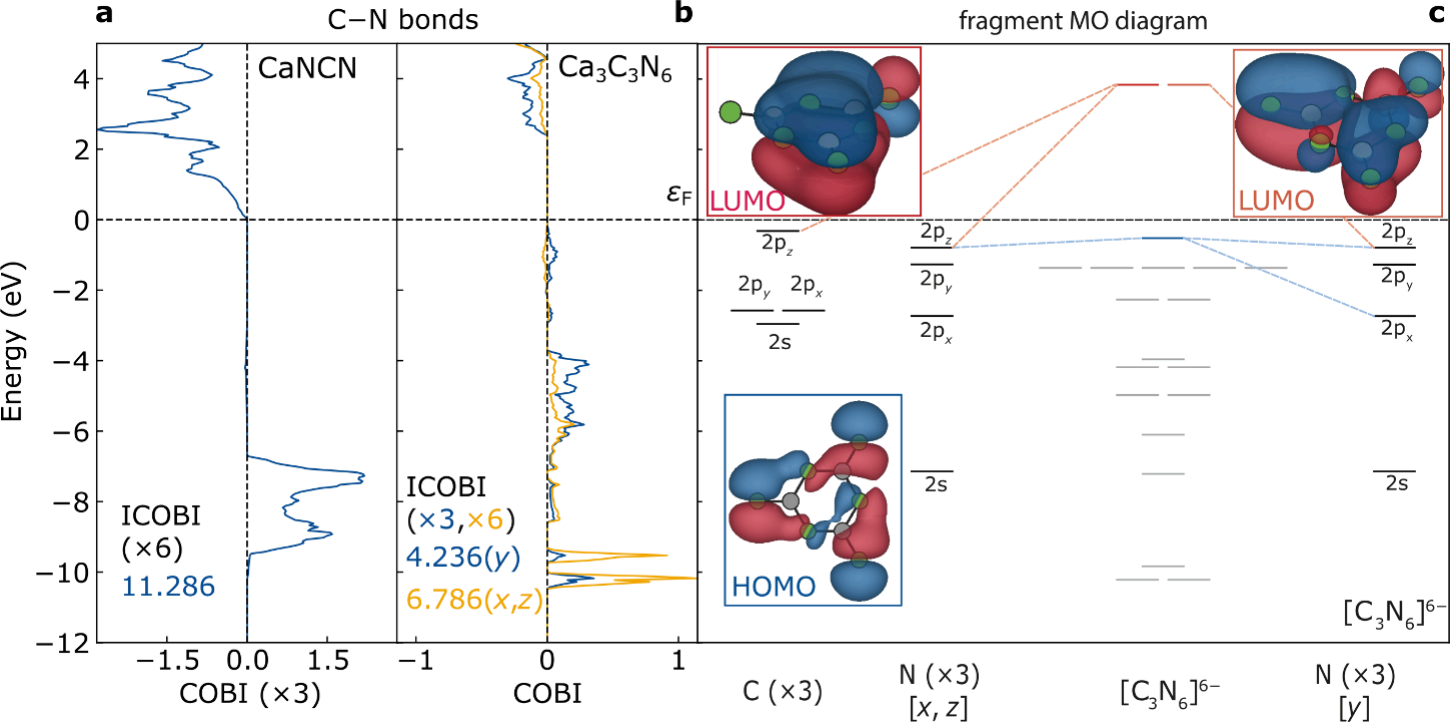
Crystal orbital bond index (COBI) analysis of C–N bonds in (a) Ca­(NCN) and (b) Ca_3_(C_3_N_6_) under a pressure of 35 GPa, with bonding interactions to the right, antibonding to the left. For easier comparison, the ICOBI of “C_3_N_6_” entity was calculated, and the ICOBI value for Ca­(NCN) was multiplied by 6 to account for the six C=N double bonds in Ca­(NCN) whereas the values for each bond (designated by *x*, *y*, and *z* in Figure 3e, respectively) in the melaminate anion were weighted by its multiplicity. (c) The fragment molecular orbital diagram of the (C_3_N_6_)^6–^ entity embedded in solid-state Ca_3_(C_3_N_6_). The MOs with energies below −12 eV or above 5 eV are omitted.

To further examine the intramolecular orbital (MO) interactions within the C_3_N_6_ entity, we generated the melaminate MO diagram from C/N atoms and their orbitals as fragments, presented in Figure 6c. The C_3_N_6_ entity in solid-state Ca_3_(C_3_N_6_) features a singly degenerate highest occupied molecular orbital (HOMO) and a set of doubly degenerate lowest unoccupied molecular orbitals (LUMOs). The HOMO consists of N 2p_*z*_ orbitals (major contributor) and N 2p_*x*_ orbitals (minor contributor) located in the deprotonated amino groups of the C_3_N_6_ entity, whereas the LUMOs are formed by 2p_*z*_ orbitals from both carbon and nitrogen atoms. There is a bonding and antibonding character in the HOMO which further cross-validates the conclusion that external pressure must compete with also populating such C_3_N_6_ antibonding interactions. Although the DFT calculations reproduce the high-pressure structural data of Cd_3_(C_3_N_6_) well (Figure 2), a slight discrepancy remains between the ambient-pressure experimental data and the DFT predictions. The latter predicts an isostructural first order phase transition at about 2 GPa (Table S11). It is likely that the transition might be kinetically hindered, and moreover, we have observed significant decrease in crystal quality for Cd_3_(C_3_N_6_) at 30 GPa, which prevented unambiguous single-crystal structure analysis below this pressure.

General crystal chemical knowledge allows us to hypothesize that melaminate salts of selected divalent metals could be thermodynamically stable and synthesizable under moderate pressure. To explore this, we performed structure optimizations for Zn- and Pb-melaminates based on the composition of M_3_(C_3_N_6_) (M = Zn, Pb). For both metals we considered three possible structure models: *hR*72 from this study as well as *hP*72 and *tP*48 reported by Ranieri et al. for Pb_3_(C_3_N_6_).^23^ For Zn_3_(C_3_N_6_) an acentric *R*3*c* structure is the most thermodynamically stable, while the reported *hP*72-Pb_3_(C_3_N_6_)^23^ and *tP*48-Pb_3_(C_3_N_6_)^23^ structures are thermodynamically more stable than in *hR*72 polymorph of Pb_3_(C_3_N_6_) featuring *R*3̅*c* space group symmetry. For the *hR*72 polymorphs, the calculations always started in the acentric *R*3*c* space group, which converged to *R*3̅*c* in the case of Pb melaminate. Calculated crystal structures and enthalpies of Zn- and Pb-melaminates are given in Tables S12–S20.

There are several strategies to direct the synthesis of hydrogen-free nitridocarbonates in a thermodynamically controlled regime in which the compounds can be produced directly from elements. The most critical factor is the choice of the countercation, stabilizing the C–N anions. For example, highly charged guanidinate anions (CN_3_)^5–^ can be stabilized by Sb^5+^ in a simple calcite-type SbCN_3_.^13^ Bigger cations like Bi^3+^/Bi^5+^ allow stabilization of polymerized C–N networks in the compound Bi_7_C_10_N_18_(N_3(1–*x*)_O_3*x*_).^36^ Pressure plays an equally important role; according to the pressure–coordination rule, carbon tends to increase its coordination number from three to four which, within valence bond theory, would be coined going from sp^2^ to sp^3^ hybridization, e.g., upon compression in carbonates.^37–41^ Binary carbon nitrides with condensed CN_4_ tetrahedra can be synthesized at pressures above 72 GPa,^42^ while pressures between 90 and 111 GPa are required for the formation of lanthanoid polynitridocarbonates MCN_3_ (M = La, Tb, Ce, Tb).^43^ The fully deprotonated form of the ortho-nitridocarbonate anion (CN_4_)^8–^ remains to be discovered but is predicted with tetravalent cations in M_2_(CN_4_) (M = Ti, Zr, Hf).^44^ The selection of suitable single-source precursors could enable the synthesis of nitridocarbonates under milder, kinetically controlled conditions, avoiding the cleavage of C–N bonds in the precursors. A notable example is the synthesis of tricyanomelaminate hydrate Na_3_C_6_N_9_·3H_2_O, featuring the (C_6_N_9_)^3–^ anion by trimerization of sodium dicyanamide NaC_2_N_3_.^45^

## Conclusions

A series of recoverable melaminate salts, M_3_(C_3_N_6_) (M = Cd, Ca), with fully deprotonated melaminate (C_3_N_6_)^6–^ was synthesized under high-pressure, high-temperature conditions. The fact that M_3_(C_3_N_6_) (M = Cd, Ca) phases were obtained with different cations and from different precursors demonstrates the high stability of such compounds and anions. While the trimerization of carbodiimide to melaminate leads to a small weakening of the C–N bonds, the M_3_(C_3_N_6_) phase mirrors an increasing Madelung field or, alternatively expressed, a more salt-like behavior upon pressure increase. Calculations also showed that the synthesis of melaminate salts might be possible with other divalent cations, e.g., zinc and lead. This opens synthetic ways to a series of inorganic nitridocarbonates with fully deprotonated melaminates by the appropriate choice of elements, even in large quantities when considering starting from single-source precursors, such as M­(NCN) (M = Cd, Ca, Zn, or Pb).

## Supplementary Material



## Notes

Overview of synthetic routes to melaminate salts; synthesis of M_3_(C_3_N_6_) (M = Cd, Ca); X-ray diffraction studies and programs; structure refinement details of M_3_(C_3_N_6_) (M = Cd, Ca); microscope picture and 2D-Raman map of M_3_(C_3_N_6_) (M = Cd, Ca); decompression by Raman of Ca_3_(C_3_N_6_); calculations of M_3_(C_3_N_6_) (M = Cd, Ca) (PDF)

## Abbreviations

LHDAC: laser-heated diamond anvil cell
DFT: density functional theory.
