# Supplementary material for: Stabilization of Fully Deprotonated Melaminate Anions (C3N6)6– in M3(C3N6) (M = Cd, Ca)
Source: J Am Chem Soc. 2026 Jan 15;148(3):2843–50. doi: 10.1021/jacs.5c16752 (PMC12856887; doi:10.1021/jacs.5c16752)
Supplement: Supplementary file 1 [file ja5c16752_si_001.pdf]

Supporting Information:

Stabilization of Fully Deprotonated Melaminate Anions $(C_3N_6)^{6-}$ in $M_3(C_3N_6)$ (M = Cd, Ca)

Pascal L. Jurzick‡[a], Lukas Brüning‡ [a], Björn Winkler[b], YiXu Wang[c], Richard Dronskowski[c], Elena Bykova[b], Dominik Spahr[b], Michael Hanfland[d], Björn Wehinger[d], Nico Giordano[e], Maxim Bykov[a]*

[a] Institute of Inorganic and Analytical Chemistry, Goethe University Frankfurt, Max-von-Laue-Straße 7, 60438 Frankfurt am Main, Germany

[b] Institute of Geosciences, Goethe University Frankfurt, Altenhoeferallee 1, 60438 Frankfurt am Main, Germany

[c] Chair of Solid-State and Quantum Chemistry, Institute of Inorganic Chemistry, RWTH Aachen University, 52056 Aachen, Germany

[d] European Synchrotron Radiation Facility, 38043 Grenoble, France

[e] Deutsches Elektronen-Synchrotron DESY, Notkestraße 85, 22607 Hamburg, Germany

Table of Contents

Section A: Overview of Synthetic Routes to Melaminate Salts 2

Section B: Synthesis of $M_3(C_3N_6)$ (M = Cd, Ca) 3

Section C: X-Ray Diffraction Studies and Programs 5

Section B: Raman Studies and Programs 6

Section D: Structure Refinement Details of $M_3(C_3N_6)$ (M = Cd, Ca) 7

Section E: Microscope Picture, 2D-Raman and 2D-PXRD Map of $Cd_3(C_3N_6)$ 10

Section F: Microscope Picture and 2D-Raman Map of $Ca_3(C_3N_6)$ 11

Section G: Decompression by Raman of $Ca_3(C_3N_6)$ 11

Section H: DFT Calculations of $M_3(C_3N_6)$ (M = Cd, Ca) 12

Section I: References 16

Section A: Overview of Synthetic Routes to Melaminate Salts

Table S1: Summary of reaction pathways for the synthesis of deprotonated melaminate anions in solid phases. Reaction products characterized *via* IR spectroscopy[a]. Reaction products characterized *via* elemental analysis[b]. Proposed reaction product[c].

Ionic Charge	No. of Hydrogen Atoms	Reaction	References
-1	5	$Na_{(s)} + C_3N_6H_{6(s)} + NH_{3(solv)}$ $\rightarrow NaC_3N_6H_5{\cdot}nNH_{3(s)}$	[1[a]]
-1	5	$K_{(s)} + C_3N_6H_{6(s)} + NH_{3(solv)}$ $\rightarrow KC_3N_6H_5{\cdot}NH_{3(s)}$	[1,2[b]]
-1	5	$Rb_{(s)} + C_3N_6H_{6(s)} + NH_{3(solv)}$ $\rightarrow RbC_3N_6H_5{\cdot}\frac{1}{2}NH_{3(s)}$	[1]
-2	4	$SbCl_{3(s)} + 3\ C_3N_6H_{6(s)}$ $\rightarrow SbCl(C_3N_6H_4)_{(s)} + 2\ (C_3N_6H_7)Cl_{(s)}$	[3]
-3	3	$3\ K_{(s)} + C_3N_6H_{6(s)} + NH_{3(solv)}$ $\rightarrow K_3(C_3N_6H_3)_{(s)}$	[1[a],2[b],4[c]]
-3	3	$3\ CuCl_{(s)} + 4\ C_3N_6H_{6(s)}$ $\rightarrow Cu_3(C_3N_6H_3)_{(s)} + 3\ (C_3N_6H_7)Cl_{(s)}$	[5]
-3	3	$3\ CuCl_{(s)} + 3\ Na(HCN_2)_{(s)}$ $\rightarrow Cu_3(C_3N_6H_3)_{(s)} + 3\ NaCl_{(s)}$	[6]
-4	2	-	-
-5	1	-	-
-6	0	$3\ Cd_{(s)} + 3\ C_{Dia(s)} + 3\ N_{2(s)}$ $\rightarrow Cd_3(C_3N_6)_{(s)}$	this work
-6	0	$6\ Cd_{(s)} + 3\ C_6N_{4(s)}$ $\rightarrow 2\ Cd_3(C_3N_6)_{(s)} + 12\ C_{(s)}$	this work
-6	0	$3\ CaCN_{2(s)} \rightarrow Ca_3(C_3N_6)_{(s)}$	this work

Section B: Synthesis of $M_3(C_3N_6)$ (M = Cd, Ca)

Two independent experiments were performed for the synthesis of $Cd_3(C_3N_6)$. In the 1st experiment, a BX90 type diamond anvil cell (DAC) equipped with single-beveled Boehler-Almax type diamonds (culet diameter size 120 µm) was used. A rhenium gasket was preindented from an initial thickness of 200 µm to a thickness of 25(3) µm. A sample cavity was laser-drilled at the center of the indentation to create a circular pressure chamber. A piece of cadmium metal (Sigma-Aldrich, ≥99%, stored in glove box) with a diameter of ~15 µm was placed in the sample cavity (60 µm in diameter) of the rhenium gasket. The sample was manipulated in a glove box filled with argon (H_2O and O_2 levels < 0.1 ppm). Nitrogen served as reactant and pressure-transmitting medium (PTM). The cadmium piece was laser-heated from one side with a Nd:YAG laser until a flash-like thermal emission was detected. Although, the emission spectrum could not be measured, our estimation based on the brightness of the heated spot suggest that maximum temperatures of ca. 2500 K were reached. The products of chemical reaction were then studied by means of synchrotron X-ray diffraction (XRD). After single-crystal structure analysis at several pressures, the cadmium metal piece was laser-heated a second time with a Nd:YAG laser until a reaction was detected by XRD. After the second heating, the pressure was determined to be 47.7(10) GPa and single-crystal structure refinement revealed the presence of $Cd_3(C_3N_6)$. The pressure was determined using the equation of state (EoS) of unreacted cadmium metal.[7] The DAC was decompressed to ambient conditions and opened in air for a few minutes before it was sealed again for XRD measurements. $Cd_3(C_3N_6)$ phase was still present in the sample chamber as evidenced by powder XRD patterns.

In the 2nd experiment, a BX90 type DAC equipped with Boehler-Almax type diamonds (culet diameter size 250 µm) was used. A piece of cadmium metal (Sigma-Aldrich, ≥99%, stored in glove box) with a diameter of ~40 µm was placed in the sample cavity (125 µm in diameter) of the rhenium gasket, which was preindented to a thickness of 25(3) µm. The sample was manipulated in a glove box filled with argon (H_2O and O_2 levels < 0.1 ppm). The sample hole was filled with C_6N_4 (Sigma-Aldrich, 96%) and compressed to 44(2) GPa. Tetracyanoethylene (C_6N_4) served as reactant and pressure-transmitting medium (PTM). The cadmium piece was laser-heated from one side with a Nd:YAG laser until a reaction was observed by XRD. After heating, the pressure decreased to 38(2) GPa and single-crystal structure refinement revealed the presence of $Cd_3(C_3N_6)$. The pressure was determined using the EoS of unreacted cadmium metal.[7] The DAC was decompressed to ambient conditions and opened in air for a few minutes before it was sealed again for XRD measurements. $Cd_3(C_3N_6)$ phase was still present in the sample chamber as evidenced by powder XRD patterns.

In a 3rd experiment, a BX90 type DAC equipped with Boehler-Almax type diamonds (culet diameter size 200 µm) was used. Calcium carbodiimide, Ca(NCN), was prepared by heating stoichiometric mixture of Ca_3N_2 and melamine at 800°C for 24 hours according to literature.[8] It was chosen as single-source precursor for loading without PTM. The compound was placed in the 100 µm hole of the rhenium gasket, which was preindented to a thickness of 25(3) µm. The sample was manipulated in a glove box filled with argon (H_2O and O_2 levels < 0.1 ppm). The sample was compressed to 34(1) GPa and laser-heated from one side for 10 min with a Coherent Diamond K-250 pulsed CO_2 laser (λ = 10600 nm). The laser power was adjusted to achieve a coupling of the laser to the sample using a laser power of 2−3 W. Focusing on the sample resulted in a heating area of ≈ 40×40 µm^2. The estimated maximum temperatures reached during the laser heating are ca. 2000 K. After the heating process, the pressure increased to 34.4(10) GPa and the structure of reaction product - $Ca_3(C_3N_6)$ was solved and refined based on single-crystal X-ray diffraction data. The pressure was determined using the shift of diamond anvil Raman peak.[9] The DAC was decompressed to ambient conditions and opened in air for 1 month before it was sealed again for XRD measurements. $Ca_3(C_3N_6)$ phase was still present in the single-crystal and powder X-ray diffraction patterns.

Section C: X-Ray Diffraction Studies and Programs

The XRD studies of LH-DAC experiments were performed at European Synchrotron Radiation Facility (ESRF) and Deutsches Elektronen-Synchrotron (DESY). The reaction products contained multiple good-quality single-crystalline domains of novel phases, which were studied by synchrotron single-crystal and powder X-ray diffraction at the beamlines ID15b (ESRF, France), ID27 (ESRF, France) and P02.2 (DESY, Germany).[10–13] The following beamline setups were used: ID15b ($\lambda \approx 0.4100$ Å, EIGER2 X 9M CdTe flat panel detector), ID27 ($\lambda \approx 0.3738$ Å, EIGER2 X 9M CdTe flat panel detector), P02.2 ($\lambda \approx 0.2908$ Å, Perkin Elmer XRD1621 flat panel detector). The beam diameters were ~2 µm (ID15b), ~1 µm (ID27), and ~3 µm (P02.2). At synthesis pressure and selected pressure points during decompression, the sample areas were scanned with the X-ray beam, resulting in 2D-PXRD grids. Powder diffraction measurements were performed without sample rotation (still images). The images were integrated to powder patterns with DIOPTAS software.[14] At selected points of the grid, single-crystal X-ray collections were collected with an angular step $\Delta\omega = 0.5°$. For the single-crystal XRD measurements the sample was rotated around vertical ω-axis in a range of ±30° or ±35°. For analysis of the single-crystal diffraction data (multigrain indexing, data integration, frame scaling and absorption correction) the CrysAlisPro software with the integrated Domain Auto Finder (DAFi) program was used.[15] To calibrate an instrumental model in the CrysAlisPro software, *e.g.* the sample-to-detector distance, detector's origin, offsets of goniometer angles, and rotation of both X-ray beam and the detector around the instrument axis we used single crystals of enstatite or vanadinite.

The crystal structures were solved by ShelXT[16] using intrinsic phasing and refined with SHELXL[17] implemented into the Olex2-1.5 program.[18] Le Bail fits of $M_3(C_3N_6)$ (M = Cd, Ca) were performed using JANA2006.[19,20] The structures were visualized with Diamond 5.0.2. program.[21] CSD 2481198-2481204 contain the supplementary crystallographic data for this paper.

Section B: Raman Studies and Programs

Raman spectroscopy measurements were performed at high pressures and at ambient conditions using an Oxford Instruments WITec alpha 300R Raman imaging microscope. The microscope was equipped with an Olympus SLMPlan N 50× objective. Measurements were performed with a 532 nm laser. We used the 1800 grooves mm^{-1} grating from the WITec UHTS 300S (VIS-NIR) spectrograph equipped with an Andor DR316B-LDC-DD CCD detector. The applied laser power was 100 mW on the sample. The spot size of the laser was ~1 µm. We assume a depth resolution of ~6 µm in the direction of the laser beam. Raman maps were measured on a grid with a step size of 1 µm. The background of the Raman spectra was corrected using the integrated WITec software package.

Section D: Structure Refinement Details of $M_3(C_3N_6)$ (M = Cd, Ca)

Table S2: Experimental single-crystal structure details of $Cd_3(C_3N_6)$ structure solutions. Cadmium atoms were refined with anisotropic displacement parameters.

Experiment	1st	1st	2nd	1st	1st
Chemical formula	$Cd_3C_3N_6$	$Cd_3C_3N_6$	$Cd_3C_3N_6$	$Cd_3C_3N_6$	$Cd_3C_3N_6$
Pressure (GPa)	47.7(10)	45.2(10)	38(2)	36.6(10)	30.0(10)
Beamline	ID15b (ESRF)	ID15b (ESRF)	ID27 (ESRF)	ID15b (ESRF)	ID15b (ESRF)
Wavelength (Å)	0.41	0.41	0.3738	0.41	0.41
Crystal sytem, space group	Trigonal, *R*3*c* (No. 161)	Trigonal, *R*3*c* (No. 161)	Trigonal, *R*3*c* (No. 161)	Trigonal, *R*3*c* (No. 161)	Trigonal, *R*3*c* (No. 161)
a, c (Å)	11.5351(12), 5.188(2)	11.528(6), 5.241(2)	11.565(4), 5.369(4)	11.563(3), 5.370(5)	11.613(2), 5.517(6)
V (Å^3)	597.9(3)	603.2(6)	621.9(6)	621.8(6)	644.3(7)
Z	6	6	6	6	6
No. of measured, independent and observed [I > 2σ(I)] reflections	481, 274, 259	445, 334, 328	365, 247, 198	539, 342, 320	528, 283, 268
R_{int}	0.010	0.009	0.023	0.023	0.021
$R[F^2 > 2\sigma(F^2)]$, $wR(F^2)$, S	0.037, 0.095, 1.08	0.042, 0.120, 1.15	0.041, 0.092, 1.02	0.057, 0.153, 1.05	0.070, 0.194, 1.11
No. of parameters	22	22	22	22	22
$\Delta\rho_{max}$, $\Delta\rho_{min}$ (e Å^{-3})	1.31, −1.73	2.54, −1.57	1.40, −1.68	2.25, −1.88	2.60, −2.15
Absolute structure parameter[a]	0.15(12)	0.33(9)	−0.06(16)	0.18(15)	0.9(3)[b]
CSD number	2481200	2481203	2481204	2481201	2481198

[a] Due to incomplete datasets caused by diamond anvil cell shadowing and high-energy radiation, the determination of absolute structure might be ambiguous.
[b] An attempt to invert the structure resulted in Hooft y: 2.70(2), Flack x: 0.1(2).

Table S3: Experimental single-crystal structure details of $Ca_3(C_3N_6)$ structure solutions. Calcium atoms were refined with anisotropic displacement parameters.

Experiment	3rd	3rd
Chemical formula	$Ca_3C_3N_6$	$Ca_3C_3N_6$
Pressure (GPa)	34.4(10)	ambient
Beamline	ID27 (ESRF)	P02.2 (DESY)
Wavelength (Å)	0.3738	0.2908
Crystal sytem, space group	Trigonal, $R\bar{3}c$ (No. 167)	Trigonal, $R\bar{3}c$ (No. 167)
a, c (Å)	11.2310(11), 5.889(8)	11.770(4), 6.721(7)
V (Å^3)	643.2(8)	806.4(11)
Z	6	6
No. of measured, independent and observed [I > 2σ(I)] reflections	314, 130, 117	656, 184, 139
R_{int}	0.019	0.067
$R[F^2 > 2\sigma(F^2)]$, $wR(F^2)$, S	0.050, 0.152, 1.22	0.077, 0.195, 1.08
No. of parameters	12	12
$\Delta\rho_{max}$, $\Delta\rho_{min}$ (e Å^{-3})	0.62, −0.54	1.24, −0.63
CSD number	2481199	2481202

Best Dataset (1st Experiment):

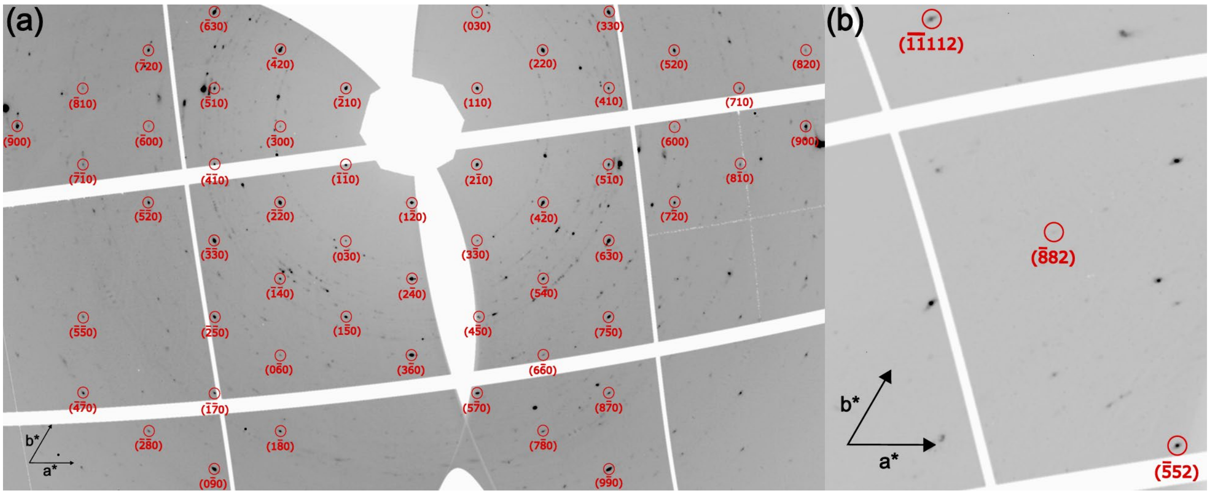

Figure S1: (a) $(hk0)$- and **(b)** $(hk2)$-reciprocal lattice plane of $Cd_3(C_3N_6)$ at 47.7(10) GPa (1st experiment) with indexed reflections. The indexed reflections in **(a)** fulfil the general condition $hkil$: $-h+k+l = 3n$ for a rhombohedral centering and in **(b)** $h\bar{h}0l$: $h+l=3n, l=2n$ for a c-glide plane perpendicular to the $[1\bar{1}0]$ direction in the hexagonal unit cell setting for rhombohedral trigonal space groups. To convert hkl to $hkil$, $i=-(h+k)$ holds.

Table S4: Fractional atomic coordinates and isotropic or equivalent displacement parameters (Å^2) of $Cd_3(C_3N_6)$ at 47.7(10) GPa (1st Experiment).

Atom	Wyckoff position	x	y	z	U_{iso}*/U_{eq}
Cd1	18b	0.00451(13)	0.45145(9)	0.65863(2)	0.0159(5)
N1	18b	0.2141(10)	0.5532(13)	0.833(5)	0.011(2)*
C1	18b	0.3261(17)	0.5484(16)	0.811(9)	0.014(2)*
N2	18b	0.1118(12)	0.4355(11)	0.358(7)	0.011(2)*

Supporting Datasets:

Table S5: Fractional atomic coordinates and isotropic or equivalent displacement parameters (Å^2) of $Cd_3(C_3N_6)$ at 45.2(10) GPa (1st Experiment).

Atom	Wyckoff position	x	y	z	U_{iso}*/U_{eq}
Cd1	18b	0.00598(12)	0.45110(11)	0.65863(2)	0.0170(4)
N1	18b	0.2134(14)	0.5539(17)	0.835(3)	0.017(2)*
C1	18b	0.3246(17)	0.5506(17)	0.826(5)	0.014(2)*
N2	18b	0.1138(14)	0.4332(14)	0.334(5)	0.020(2)*

Table S6: Fractional atomic coordinates and isotropic or equivalent displacement parameters (Å^2) of $Cd_3(C_3N_6)$ at 36.6(10) GPa (1st Experiment).

Atom	Wyckoff position	x	y	z	U_{iso}*/U_{eq}
Cd1	18b	0.00952(13)	0.45041(13)	0.65863(2)	0.0145(5)
N1	18b	0.2106(18)	0.553(2)	0.839(5)	0.015(3)*
C1	18b	0.3210(18)	0.547(2)	0.837(6)	0.013(3)*
N2	18b	0.1172(16)	0.4293(15)	0.334(6)	0.012(3)*

Table S7: Fractional atomic coordinates and isotropic or equivalent displacement parameters (Å^2) of $Cd_3(C_3N_6)$ at 30.0(10) GPa (1st Experiment).

**Atom**	**Wyckoff position**	***x***	***y***	***z***	U_{iso}*/U_{eq}
Cd1	18*b*	0.0121(2)	0.4489(2)	0.65863(2)	0.0228(13)
N1	18*b*	0.211(3)	0.557(3)	0.832(11)	0.024(6)*
C1	18*b*	0.317(3)	0.546(3)	0.824(16)	0.022(5)*
N2	18*b*	0.118(3)	0.427(3)	0.341(14)	0.024(5)*

Table S8: Fractional atomic coordinates and isotropic or equivalent displacement parameters (Å^2) of $Cd_3(C_3N_6)$ at 38.0(10) GPa (2nd Experiment).

**Atom**	**Wyckoff position**	***x***	***y***	***z***	U_{iso}*/U_{eq}
Cd1	18*b*	0.00939(14)	0.45002(16)	0.65863(2)	0.0141(4)
N1	18*b*	0.2110(16)	0.5556(16)	0.837(4)	0.010(3)*
C1	18*b*	0.3224(19)	0.5462(19)	0.827(7)	0.012(3)*
N2	18*b*	0.1164(18)	0.4264(16)	0.342(6)	0.016(3)*

Table S9: Fractional atomic coordinates and isotropic or equivalent displacement parameters (Å^2) of $Ca_3(C_3N_6)$ at 34.4(10) GPa (3rd Experiment).

**Atom**	**Wyckoff position**	***x***	***y***	***z***	U_{iso}*/U_{eq}
Ca1	18*e*	0.6667	0.78313(9)	0.5833	0.0368(17)
N1	18*e*	0.4542(4)	0.6667	0.4167	0.0210(10)*
C1	18*e*	0.3333	0.5467(4)	0.4167	0.0201(11)*
N2	18*e*	0.3333	0.4307(4)	0.4167	0.0211(10)*

Table S10: Fractional atomic coordinates and isotropic or equivalent displacement parameters (Å^2) of $Ca_3(C_3N_6)$ at ambient pressure (3rd Experiment).

**Atom**	**Wyckoff position**	***x***	***y***	***z***	U_{iso}*/U_{eq}
Ca1	18*e*	0.6667	0.88561(10)	0.5833	0.0296(10)
N1	18*e*	0.4530(5)	0.7863(5)	0.4167	0.0263(15)*
C1	18*e*	0.3333	0.7843(7)	0.4167	0.0295(18)*
N2	18*e*	0.3333	0.8972(5)	0.4167	0.0279(15)*

Section E: Microscope Picture, 2D-Raman and 2D-PXRD Map of $Cd_3(C_3N_6)$

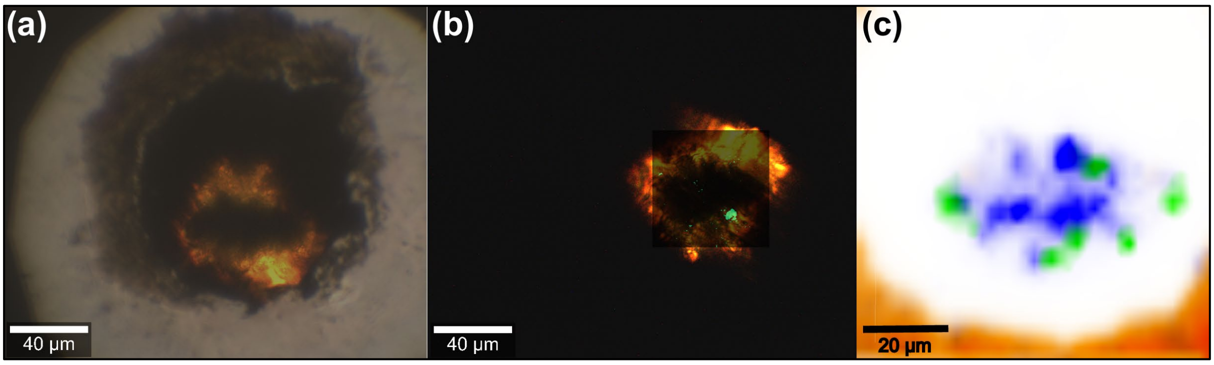

Figure S2: (a) Microscope picture of sample chamber after heating cadmium metal piece in tetracyanoethylene (C_6N_4) with Nd:YAG laser. **(b)** 2D-Raman map after synthesis of $Cd_3(C_3N_6)$ (2nd experiment, 38(2) GPa). Green color code corresponds to a Raman band at 1588 cm^{-1} of $Cd_3(C_3N_6)$. Orange color appears due to transmission of light through semi-transparent tetracyanoethylene. **(c)** 2D-PXRD map after synthesis of $Cd_3(C_3N_6)$ (2nd experiment, 38(2) GPa). Green color code corresponds to intensity of [6$\bar{3}$0] reflection of $Cd_3(C_3N_6)$. Blue color code corresponds to the intensity of [101] reflection of recrystallized cadmium metal. Orange color code corresponds to the intensity of [101] reflection of rhenium metal gasket.

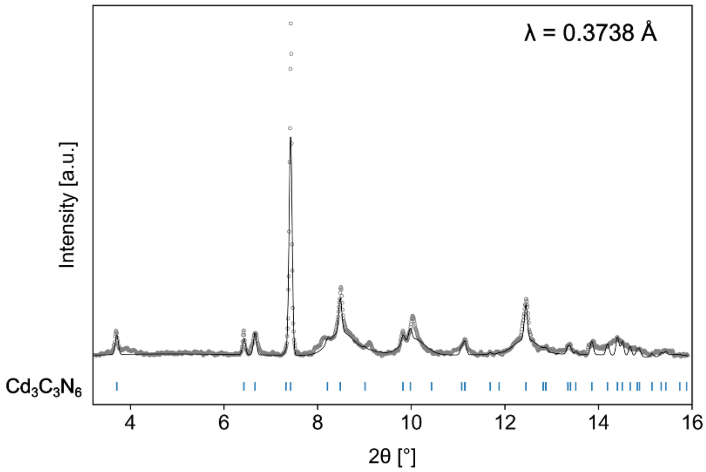

Figure S3: Powder X-ray diffraction pattern and Le Bail fit from multigrain $Cd_3(C_3N_6)$ at ambient conditions. The broad peaks (*e.g.* at 8.5 °) are likely related to poorly-crystallized C–N containing phase originating from the C_6N_4 precursor.

Section F: Microscope Picture and 2D-Raman Map of $Ca_3(C_3N_6)$

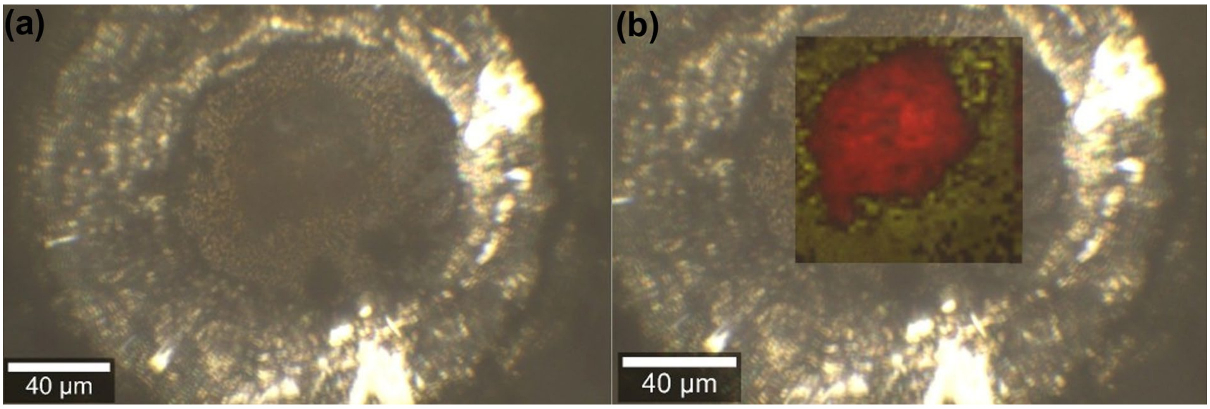

Figure S4: (a) Microscope picture of sample chamber with $CaCN_2$ after heating with CO_2 laser and **(b)** 2D-Raman map after synthesis of $Ca_3(C_3N_6)$ (3rd experiment, 34.4(10) GPa). Red color code corresponds to a Raman band at 785 cm^{-1} of $Ca_3(C_3N_6)$. Yellow color code corresponds to unreacted Ca(NCN).

Section G: Decompression by Raman of $Ca_3(C_3N_6)$

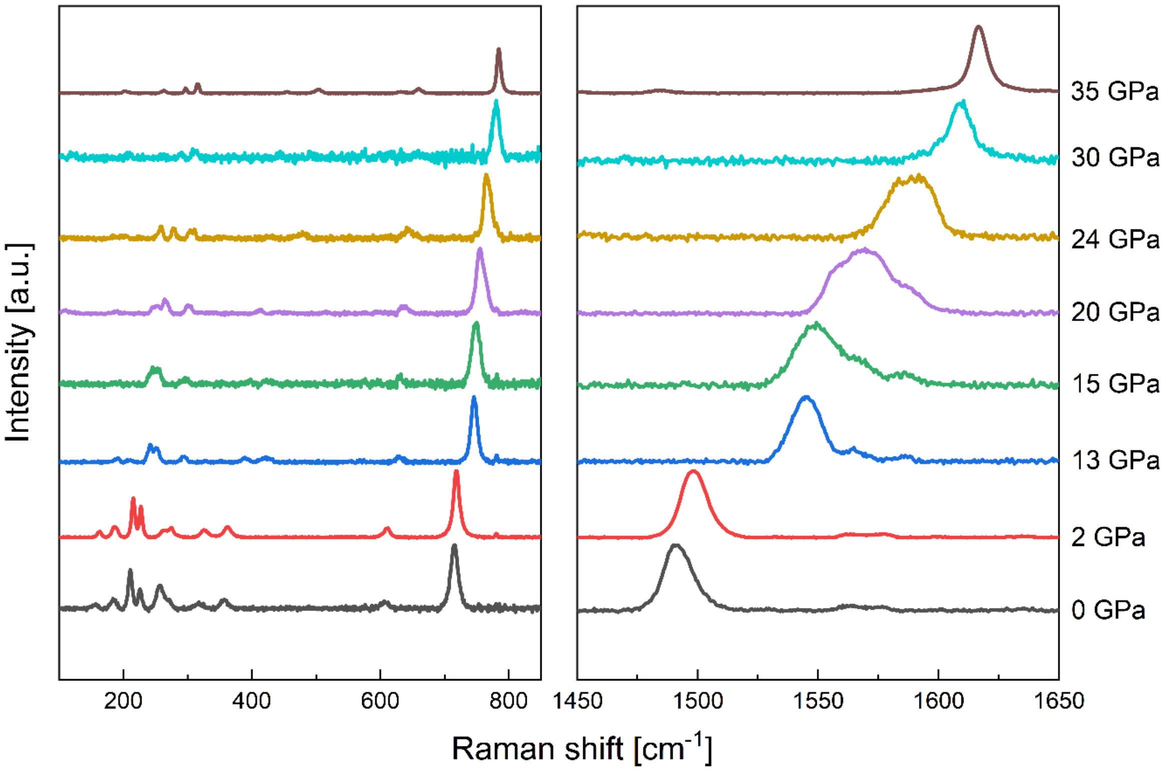

Figure S5: Raman spectra of $Ca_3(C_3N_6)$ upon decompression (3rd experiment).

Section H: DFT Calculations of $M_3(C_3N_6)$ (M = Cd, Ca)

First-principles calculations were carried out within the framework of density functional theory (DFT), employing the Perdew-Burke-Ernzerhof (PBE) exchange-correlation functional and the plane wave/pseudopotential approach implemented in the CASTEP simulation package.[22–24] "On the fly" norm-conserving pseudopotentials generated using the descriptors in the CASTEP data base were employed in conjunction with plane waves up to a kinetic energy cutoff of 1000eV. The accuracy of the pseudopotentials is well established.[25] A Monkhorst-Pack grid was used for Brillouin zone integrations.[26] We used a distance between grid points of <0.023 $Å^{-1}$. Convergence criteria for geometry optimization included an energy change of <5 x 10^{-6} eV $atom^{-1}$ between steps, a maximal force of <0.008 eV $Å^{-1}$ and a maximal component of the stress tensor <0.02 GPa. Phonon frequencies were obtained from density functional perturbation theory (DFPT) calculations.[27,28] Raman intensities were computed using DFPT with the "2n + 1" theorem approach.[29]

The DFT chemical-bonding calculations of $Ca_3(C_3N_6)$ (and related CaNCN for comparison) were carried out using the Vienna ab initio simulation package (VASP, version 6.1.1).[30,31] Pseudopotentials following projector augmented wave (PAW) methodology and Perdew–Burke–Ernzerhof (PBE) parameterization of generalized gradient approximation (GGA) exchange-correlation functionals were used.[23,32] The energy cutoff was set to be 800 eV and Γ-centered Monkhorst-Pack *k* point meshes were automatically generated with a *k* point density of 2π × 0.02 $Å^{-1}$ for sampling the first Brillouin zone. Geometry optimization was conducted prior to accurate self-consistent field (SCF) calculations. The convergence criteria were 10^{-8} eV for energy (for both geometry optimization and SCF calculations) and 10^{-6} eV/Å for forces (only for geometry optimization). Gaussian smearing together with a 0.02 eV smearing width (for structural optimization) and tetrahedron method with Blöchl corrections (for accurate energy calculations) were chosen for integration over *k* points. Based on wavefunction information obtained from plane wave theory, a unitary transformation in reciprocal space was performed by the Local Orbital Basis Suite Towards Electronic Structure Reconstruction (LOBSTER, version 5.1.1) code to extract chemical bonding information based on localized atomic orbitals.[33,34] The chemical bonding analysis was postprocessed and visualized using LOPOSTER.[35]

Table S11: Fractional atomic coordinates of *hR*72-$Cd_3(C_3N_6)$ polymorph in *R*3*c* at 0 GPa (DFT Model).

Atom	Wyckoff position	*x*	*y*	*z*
Cd1	18*b*	−0.05569	−0.30223	−0.54844
N1	18*b*	−0.19894	−0.56135	−0.40746
C1	18*b*	−0.30507	−0.53404	−0.42481
N2	18*b*	−0.27314	−0.40776	−0.47113

Table S12: Structural details of $Pb_3(C_3N_6)$ polymorphs (DFT Model).

**Compounds**	***hR*72-$Pb_3(C_3N_6)$**	***tP*48-$Pb_3(C_3N_6)$**	***hP*72-$Pb_3(C_3N_6)$**
Chemical formula	$Pb_3C_3N_6$	$Pb_3C_3N_6$	$Pb_3C_3N_6$
Pressure (GPa)	45	45	45
Crystal sytem, space group	Trigonal, $R\bar{3}c$ (No. 167)	Tetragonal, $P\bar{4}2_1m$(No. 113)	Hexagonal, $P6_522$ (No. 179)
a, *c* (Å)	12.0699, 5.7421	11.9110, 3.2901	7.4853, 14.0601
V ($Å^3$)	724.45	466.77	682.24

Table S13: Fractional atomic coordinates of *hR*72-$Pb_3(C_3N_6)$ in $R\bar{3}c$ at 45 GPa (DFT Model).

Atom	Wyckoff position	*x*	*y*	*z*
Pb1	18*e*	0.33333	0.21370	−0.58333
N1	18*e*	0.55501	0.33333	−0.41667
C1	18*e*	0.55657	0.22323	−0.41667
N2	18*e*	0.44731	0.11398	−0.41667

Table S14: Fractional atomic coordinates of *tP*48-$Pb_3(C_3N_6)$ in $P\bar{4}2_1m$ at 45 GPa (DFT Model).

Atom	Wyckoff position	*x*	*y*	*z*
Pb1	8*f*	0.08188	0.13826	0.71969
N3	8*f*	0.03620	0.26545	0.18643
N4	8*f*	0.12310	0.76265	0.80937
C2	8*f*	0.06422	0.69991	0.06971
Pb2	4*e*	0.08926	0.41074	0.60673
N2	4*e*	0.22543	0.27457	0.61723
N1	4*e*	0.09909	0.59909	0.19577
C1	4*e*	0.22319	0.72319	0.66960

Table S15: Fractional atomic coordinates of *hP*72-$Pb_3(C_3N_6)$ in $P6_522$ at 45 GPa (DFT Model).

Atom	Wyckoff position	*x*	*y*	*z*
Pb1	12*c*	0.19902	0.39945	0.88697
N3	12*c*	0.07572	0.53931	0.75749
N4	12*c*	0.08117	0.22894	0.07393
C2	12*c*	0.02221	0.35461	0.25464
Pb2	6*b*	0.19815	0.59908	0.41667
N1	6*b*	0.23049	0.46098	0.25000
C1	6*b*	0.02150	0.04301	0.25000
N2	6*b*	0.08088	0.16176	0.75000

Table S16: Structural details of $Zn_3(C_3N_6)$ polymorphs (DFT Model).

Compounds	*hR*72-$Zn_3(C_3N_6)$	*tP*48-$Zn_3(C_3N_6)$	*hP*72-$Zn_3(C_3N_6)$
Chemical formula	$Zn_3C_3N_6$	$Zn_3C_3N_6$	$Zn_3C_3N_6$
Pressure (GPa)	45	45	45
Crystal system, space group	Trigonal, *R*3*c* (No. 161)	Tetragonal, $P\bar{4}2_1m$(No. 113)	Hexagonal, $P6_522$ (No. 179)
a, c (Å)	10.9621, 5.1330	11.2357, 2.8999	7.3650, 11.8550
V (Å^3)	534.18	366.09	556.90

Table S17: Fractional atomic coordinates of *hR*72-$Zn_3(C_3N_6)$ in $R3c$ at 45 GPa (DFT Model).

Atom	Wyckoff position	*x*	*y*	*z*
Zn1	18*b*	−0.10177	−0.35374	−0.59665
N1	18*b*	−0.22815	−0.53309	−0.43215
C1	18*b*	−0.36183	−0.56168	−0.42830
N2	18*b*	−0.39342	−0.46182	−0.39475

Table S18: Fractional atomic coordinates of *tP*48-$Zn_3(C_3N_6)$ in $P\bar{4}2_1m$ at 45 GPa (DFT Model).

Atom	Wyckoff position	*x*	*y*	*z*
Zn1	8*f*	0.08022	0.09511	0.76120
N3	8*f*	0.01124	0.23726	0.00944
N4	8*f*	0.15843	0.79982	0.62787
C2	8*f*	0.07915	0.72044	−0.20610
Zn2	4*e*	0.07681	0.42319	0.11924
N2	4*e*	0.15274	0.34726	0.61139
N1	4*e*	0.10061	0.60061	−0.20482
C1	4*e*	0.27078	0.77078	0.53333

Table S19: Fractional atomic coordinates of *hP*72-$Zn_3(C_3N_6)$ in $P6_522$ at 45 GPa (DFT Model).

Atom	Wyckoff position	*x*	*y*	*z*
Zn1	12*c*	0.11514	0.30123	0.89985
N3	12*c*	0.00950	0.39535	0.78194
N4	12*c*	0.00314	0.30246	0.05020
C2	12*c*	0.09944	0.50805	0.27982
Zn2	6*b*	0.39047	0.69523	0.41667
N1	6*b*	0.30912	0.61823	0.25000
C1	6*b*	0.10104	0.20207	0.25000
N2	6*b*	0.00162	0.00324	0.75000

Table S20: Calculated enthalpies of different $M_3C_3N_6$ (*M*= Zn, Pb) polymorphs at 45 GPa relative to *hR*72 polymorphic modification. Enthalpies are given in kJ/mol.

$Zn_3C_3N_6$ (45 GPa)	*hR*72 ($R3c$) Z=6	*hP*72 Z=6	*tP*48 Z=4
Δ*H*	0	+287.5	+457.6
$Pb_3C_3N_6$ (45 GPa)	*hR*72 (R-3c) Z=6	*hP*72 Z=6	*tP*48 Z=4
Δ*H*	0	-102.1	-108.9

Table S21: Wavefunction-based Löwdin charges (unit: *e*) of Ca(NCN) and $Ca_3(C_3N_6)$ obtained from LOBSTER.

Atom	Ca(NCN) (0 GPa)	Ca(NCN) (35 GPa)	$Ca_3(C_3N_6)$ (35 GPa)
Ca	+1.66	+1.63	+1.60
C	+0.20	+0.22	+0.23
N	−0.93	−0.92	−0.64 (*y*) / −1.19(*x*, *z*)

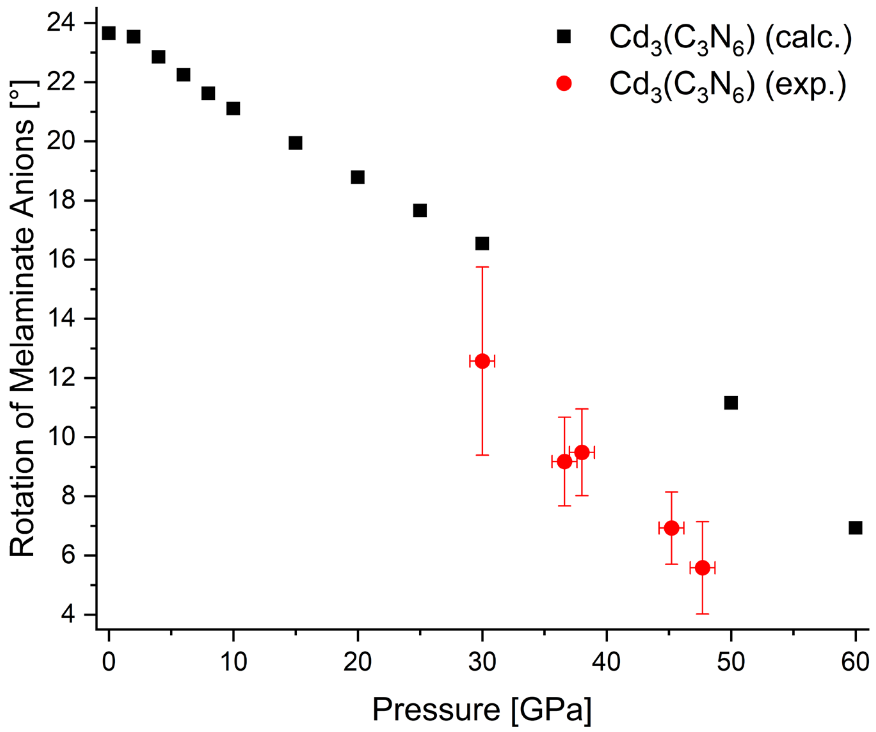

Figure S6: Rotation of melaminate anions with respect to each other in the neighboring layers of $Cd_3(C_3N_6)$ upon decompression. Experimental data (red) and DFT calculated data (black).

Section I: References

[1] A. L. Görne, T. Scholz, D. Kobertz, R. Dronskowski, "Deprotonating Melamine to Gain Highly Interconnected Materials: Melaminate Salts of Potassium and Rubidium" *Inorg. Chem.* **2021**, *60*, 15069–15077.

[2] E. C. Franklin, "THE AMMONO CARBONIC ACIDS" *J. Am. Chem. Soc.* **1922**, *44*, 486–509.

[3] E. Bayat, M. Ströbele, H.-J. Meyer, "Unraveling the Synthesis of $SbCl(C_3N_6H_4)$: A Metal-Melaminate Obtained through Deprotonation of Melamine with Antimony(III)Chloride" *Chemistry* **2023**, *5*, 1465–1476.

[4] W. Schnick, H. Huppertz, "Darstellung, Kristallstruktur und Eigenschaften von Kaliumhydrogencyanamid" *Z. Anorg. Allg. Chem.* **1995**, *621*, 1703–1707.

[5] P. Kallenbach, E. Bayat, M. Ströbele, C. P. Romao, H.-J. Meyer, "Tricopper Melaminate, a Metal–Organic Framework Containing Dehydrogenated Melamine and Cu–Cu Bonding" *Inorg. Chem.* **2021**, *60*, 16303–16307.

[6] E. Bayat, M. Ströbele, M. Abbasi, S. Kroeker, J. Valenta, D. Enseling, T. Jüstel, H.-J. Meyer, "High-Yield Synthesis Route, Post-Synthesis Treatment, and Insights into the Photoluminescence and Magnetic Properties of Tricopper(I) Melaminate $Cu_3(C_3N_6H_3)$" *Inorg. Chem.* **2024**, *63*, 19053–19062.

[7] T. Kenichi, "Structural study of Zn and Cd to ultrahigh pressures" *Phys. Rev. B* **1997**, *56*, 5170–5179.

[8] U. Berger, W. Schnick, "Syntheses, crystal structures, and vibrational spectroscopic properties of $MgCN_2$, $SrCN_2$, and $BaCN_2$" *J. Alloys Compd.* **1994**, *206*, 179–184.

[9] Y. Akahama, H. Kawamura, "Pressure calibration of diamond anvil Raman gauge to 310GPa" *J. Appl. Phys.* **2006**, *100*, 043516.

[10] G. Garbarino, M. E. Hanfland, S. Gallego-Parra, A. D. Rosa, M. Mezouar, D. Duran, K. Martel, E. Papillon, T. Roth, P. Got, Jer. Jacobs, "Extreme conditions X-ray diffraction and imaging beamline ID15B on the ESRF extremely brilliant source" *High Press. Res.* **2024**, *44*, 199–216.

[11] M. Mezouar, W. A. Crichton, S. Bauchau, F. Thurel, H. Witsch, F. Torrecillas, G. Blattmann, P. Marion, Y. Dabin, J. Chavanne, O. Hignette, C. Morawe, C. Borel, "Development of a new state-of-the-art beamline optimized for monochromatic single-crystal and powder X-ray diffraction under extreme conditions at the ESRF" *J. Synchrotron Radiat.* **2005**, *12*, 659–664.

[12] S. Petitgirard, A. Salamat, P. Beck, G. Weck, P. Bouvier, "Strategies for *in situ* laser heating in the diamond anvil cell at an X-ray diffraction beamline" *J. Synchrotron Radiat.* **2014**, *21*, 89–96.

[13] H.-P. Liermann, Z. Konôpková, W. Morgenroth, K. Glazyrin, J. Bednarčik, E. E. McBride, S. Petitgirard, J. T. Delitz, M. Wendt, Y. Bican, A. Ehnes, I. Schwark, A. Rothkirch, M. Tischer, J. Heuer, H. Schulte-Schrepping, T. Kracht, H. Franz, "The Extreme Conditions Beamline P02.2 and the Extreme Conditions Science Infrastructure at PETRA III" *J. Synchrotron Radiat.* **2015**, *22*, 908–924.

[14] C. Prescher, V. B. Prakapenka, "*DIOPTAS* : a program for reduction of two-dimensional X-ray diffraction data and data exploration" *High Press. Res.* **2015**, *35*, 223–230.

[15] A. Aslandukov, M. Aslandukov, N. Dubrovinskaia, L. Dubrovinsky, "*Domain Auto Finder* (*DAFi*) program: the analysis of single-crystal X-ray diffraction data from polycrystalline samples" *J. Appl. Crystallogr.* **2022**, *55*, 1383–1391.

[16] G. M. Sheldrick, "*SHELXT* – Integrated space-group and crystal-structure determination" *Acta Cryst.* **2015**, *A71*, 3–8.

[17] G. M. Sheldrick, "Crystal structure refinement with *SHELXL*" *Acta Cryst.* **2015**, *71*, 3–8.

[18] O. V. Dolomanov, L. J. Bourhis, R. J. Gildea, J. A. K. Howard, H. Puschmann, "*OLEX2* : a complete structure solution, refinement and analysis program" *J. Appl. Crystallogr.* **2009**, *42*, 339–341.

[19] A. Le Bail, "Whole powder pattern decomposition methods and applications: A retrospection" *Powder Diffr.* **2005**, *20*, 316–326.

[20] V. Petříček, M. Dušek, L. Palatinus, “Crystallographic Computing System JANA2006: General features” *Z. Kristallogr. – Cryst. Mater.* **2014**, *229*, 345–352.
[21] G. Bergerhoff, M. Berndt, K. Brandenburg, “Evaluation of crystallographic data with the program DIAMOND” *J. Res. Natl. Inst. Stand. Technol.* **1996**, *101*, 221.
[22] P. Hohenberg, W. Kohn, “Inhomogeneous Electron Gas” *Phys. Rev.* **1964**, *136*, B864–B871.
[23] J. P. Perdew, K. Burke, M. Ernzerhof, “Generalized Gradient Approximation Made Simple” *Phys. Rev. Lett.* **1996**, *77*, 3865–3868.
[24] S. J. Clark, M. D. Segall, C. J. Pickard, P. J. Hasnip, M. I. J. Probert, K. Refson, M. C. Payne, “First principles methods using CASTEP” *Z. Kristallogr.* **2005**, *220*, 567–570.
[25] K. Lejaeghere, G. Bihlmayer, T. Björkman, P. Blaha, S. Blügel, V. Blum, D. Caliste, I. E. Castelli, S. J. Clark, A. Dal Corso, S. De Gironcoli, T. Deutsch, J. K. Dewhurst, I. Di Marco, C. Draxl, M. Dułak, O. Eriksson, J. A. Flores-Livas, K. F. Garrity, L. Genovese, P. Giannozzi, M. Giantomassi, S. Goedecker, X. Gonze, O. Grånäs, E. K. U. Gross, A. Gulans, F. Gygi, D. R. Hamann, P. J. Hasnip, N. A. W. Holzwarth, D. Iuşan, D. B. Jochym, F. Jollet, D. Jones, G. Kresse, K. Koepernik, E. Küçükbenli, Y. O. Kvashnin, I. L. M. Locht, S. Lubeck, M. Marsman, N. Marzari, U. Nitzsche, L. Nordström, T. Ozaki, L. Paulatto, C. J. Pickard, W. Poelmans, M. I. J. Probert, K. Refson, M. Richter, G.-M. Rignanese, S. Saha, M. Scheffler, M. Schlipf, K. Schwarz, S. Sharma, F. Tavazza, P. Thunström, A. Tkatchenko, M. Torrent, D. Vanderbilt, M. J. Van Setten, V. Van Speybroeck, J. M. Wills, J. R. Yates, G.-X. Zhang, S. Cottenier, “Reproducibility in density functional theory calculations of solids” *Science* **2016**, *351*, aad3000.
[26] H. J. Monkhorst, J. D. Pack, “Special points for Brillouin-zone integrations” *Phys. Rev. B* **1976**, *13*, 5188.
[27] S. Baroni, S. De Gironcoli, A. Dal Corso, P. Giannozzi, “Phonons and related crystal properties from density-functional perturbation theory” *Rev. Mod. Phys.* **2001**, *73*, 515–562.
[28] K. Refson, P. R. Tulip, S. J. Clark, “Variational density-functional perturbation theory for dielectrics and lattice dynamics” *Phys. Rev. B* **2006**, *73*, 155114.
[29] K. Miwa, “Prediction of Raman spectra with ultrasoft pseudopotentials” *Phys. Rev. B* **2011**, *84*, 094304.
[30] G. Kresse, J. Furthmüller, “Efficient iterative schemes for *ab initio* total-energy calculations using a plane-wave basis set” *Phys. Rev. B* **1996**, *54*, 11169–11186.
[31] G. Kresse, J. Furthmüller, “Efficiency of ab-initio total energy calculations for metals and semiconductors using a plane-wave basis set” *Comput. Mater. Sci.* **1996**, *6*, 15–50.
[32] J. P. Perdew, J. A. Chevary, S. H. Vosko, K. A. Jackson, M. R. Pederson, D. J. Singh, C. Fiolhais, “Atoms, molecules, solids, and surfaces: Applications of the generalized gradient approximation for exchange and correlation” *Phys. Rev. B* **1992**, *46*, 6671–6687.
[33] S. Maintz, V. L. Deringer, A. L. Tchougréeff, R. Dronskowski, "LOBSTER: A tool to extract chemical bonding from plane-wave based DFT" *J. Comput. Chem.* **2016**, *37*, 1030–1035.
[34] R. Nelson, C. Ertural, J. George, V. L. Deringer, G. Hautier, R. Dronskowski, “LOBSTER : Local orbital projections, atomic charges, and chemical-bonding analysis from PROJECTOR-AUGMENTED-WAVE-BASED density-functional theory" *J. Comput. Chem.* **2020**, *41*, 1931–1940.
[35] Y. Wang, P. C. Müller, D. Hemker, R. Dronskowski, “LOPOSTER : A Cascading Postprocessor for LOBSTER” *J. Comput. Chem.* **2025**, *46*, e70167.
